# Drug repurposing against fucosyltransferase-2 *via* docking, STD-NMR, and molecular dynamic simulation studies

**DOI:** 10.1371/journal.pone.0308517

**Published:** 2024-11-01

**Authors:** Muhammad Atif, Humaira Zafar, Atia-tul- Wahab, M. Iqbal Choudhary

**Affiliations:** 1 International Center for Chemical and Biological Sciences, H. E. J. Research Institute of Chemistry, University of Karachi, Karachi, Pakistan; 2 Dr. Panjwani Center for Molecular Medicine and Drug Research, International Center for Chemical and Biological Sciences, University of Karachi, Karachi, Pakistan; 3 Department of Biochemistry, Faculty of Science, King Abdulaziz University, Jeddah, Saudi Arabia; PhD, Concordia University, CANADA

## Abstract

Aberrant fucosylation is the hallmark of malignant cell transformation, leading to many cellular events, such as uncontrolled cell proliferation, angiogenesis, tumor cell invasion, and metastasis. This increased fucosylation is caused due to the over-expression of fucosyltransferases (FUTs) that catalyzes the transfer of the fucose (Fuc) residue from GDP-fucose (donor substrate) to various oligosaccharides, glycoproteins, and glycolipids (acceptor substrates). Hence, fucosyltransferases (FUTs) are considered as validated target for the drug discovery against on cancers. In the current study, a drug repurposing approach was deployed to identify new hits against fucosyltransferase 2 (FUT2), using computational and biophysical techniques. A library of 500 US-FDA approved drugs were screened *in-silico* against fucosyltransferase 2 (FUT2) donor and acceptor sites. Five drugs were predicted as hits, based on their significant docking scores (-5.8 to -8.2), and binding energies (-43 to -51.19 Kcal/mol). Furthermore, STD-NMR highlighted the epitope of these drugs in the binding site of fucosyltransferase 2 (FUT2). Simulation studies provided insights about the binding site of these drugs, and 4 of them, acarbose, ascorbic acid, ibuprofen, and enalaprilat dihydrate, were found as significant binders at the donor binding site of fucosyltransferase 2 (FUT2). Hence, the current study reports the repurposed drugs as potential hits against fucosyltransferase 2 (FUT2). These may be further studied through *in-vitro* and *in-vivo* inhibitory and mechanistic studies.

## 1. Introduction

Drug discovery is a time, and cost-intensive process. Drug repurposing thus emerged as a popular strategy for reducing the overall cost and time required for drugs development in recent years [1]. The goal of drug repurposing is to identify a new indication for an existing US FDA-approved drugs [2]. Considering the above-mentioned advantages, the current research was aimed to identify the potent inhibitors of the clinically important enzyme fucosyltransferase 2 (FUT2), using the drug repurposing approach.

Fucosyltransferases (FUTs) are the bi-substrate enzymes that use GDP-fucose as a donor substrate and catalyzes the transfer of fucose moiety to various sugar acceptors [3]. Among various fucosyltransferases, fucosyltransferases 2 (FUT2) is responsible for the production of A, B, and H antigens, as well as α-1,2-linked fucosylated glycans (Globo H, and Lewis^y^, are glycan products of 1, 2-fucosyltransferases, which are highly expressed on malignant tissues). The over-production of these glycans have reportedly been found in a variety of malignancies, such as breast, colon, and lungs cancers [4, 5].

Aberrant expression of fucosylated glycoconjugates is associated with cell adhesion and metastasis, thereby promoting tumor progression. Fucosylation is among the key post-translation modifications required for cell growth and survival [1]. Overexpression of fucosyltransferases (FUTs) leads to increased fucosylated glycans that help in the development and progression of cancers [2]. Hence, fucosyltransferases (FUTs) are considered as valid targets for cancer drug discovery [3].

Various approaches have been employed for the development of fucosyltransferases (FUTs) inhibitors, including the design of substrate or transition state analogues through chemical synthesis [6]. However, till date, there is no clinical drug available against any fucosyltransferase (FUT) enzyme. There are various reasons for the limited success in the development of fucosyltransferases (FUTs). A few limited studies on crystal structures of complex donor and acceptor substrates, as well as relatively complex biochemical assays system are among the key reasons. Hence, the current study was an effort to use computational and biophysical approches to identify potential binders against fucosyltransferase 2 (FUT2) [7, 8].

## 2. Material and methods

### 2.1 Chemicals

Guanosine-5′-diphosphate-β-L-fucose sodium salt (Cat. No. G4401), *N*-acetyl-D-lactosamine (Cat. No. A7791), DMSO HPLC grade (Cat. No. 34869–2.5L), *Tris* (hydroxymethyl) aminomethane (Cat. No. 106B), hydrochloric acid (Cat. No. H1758), *N*-ethyl maleimide (Cat. No. E3876), deuterium oxide (Cat. No. 014100.2050), deuterated tris (Cat. No. DLM- 1814–5), and deuterated DMSO (Cat. No. 015100.2040) were purchased from Armar Chemicals, Switzerland. Recombinant fucosyltransferase 2 (FUT2) (Cat. No. RPF192Hu01) was purchased from Cloud-Clone Corp. (CCC, USA). Drugs included acarbose, ascorbic acid, ibuprofen, enalaprilat dihydrate and ceftriaxone sodium were available at the Dr. Panjwani Center for Molecular Medicine at Drug Research (PCMD).

### 2.2 Homology modeling

#### 2.2.1 Homology search and selection of target proteins

For the homology modeling, we performed the sequence similarity search of fucosyltransferase 2 (FUT2) with the available PDB structures using BLAST tool [9]. There is no sequence available to produce significant alignment with *E* value better than threshold. Therefore, we performed the selected homology search of fucosyltransferase 2 (FUT2) with *N*-acetyllactosamine (LacNAc), and GDP-fucose binding proteins. For this purpose, we initially searched the PDB for *N*-acetyllactosamine (LacNAc) binding proteins. Six *N*-acetyllactosamine (LacNAc) binding proteins were found, and BLAST was performed for each of these proteins with the fucosyltransferase 2 (FUT2) sequence. Similarly, GDP-fucose binding proteins were searched in PDB, and five proteins with GDP-fucose as ligand in their active site were found. BLAST was run with each of these proteins individually. None of these proteins showed more than 50% sequence similarity with fucosyltransferase 2 (FUT2), so we analyzed the results of BLAST for each protein individually. The aim was to look for the amino acids involved in the binding with their respective ligands *N*-acetyllactosamine (LacNAc), and GDP-fucose (guanosine diphosphate fucose). We observed that in case of acceptor binding site *N*-acetyllactosamine (LacNAc), the amino acids that showed the highest similarity with fucosyltransferase 2 (FUT2) were from hemolytic lectin LSLa of mushroom *Laetiporus sulphureus* complexed, with *N*-acetyllactosamine (PDB ID: 1W3F) [10]. While in case of donor binding site (GDP-fucose), the highest similarity was observed with the crystal structure of GDP-fucose protein *O*-fucosyltransferase 1 (POFUT1), in complex with GDP-fucose (crystal-form-I) (PDB ID: 3ZY5) [11, 12].

#### 2.2.2 Homology model for fucosyltransferase 2 (FUT2)

Crystal structure of hemolytic lectin (LSLa) from the mushroom *Laetiporus sulphureus*, complexed with *N*-acetyllactosamine (PDB ID: 1W3F) was used for *in-silico* modelling of acceptor binding domain of fucosyltransferase 2 (FUT2), while the crystal structure of protein *O*-fucosyltransferase 1 (POFUT1) from *Caenorhabditis elegans*, in complex with GDP-fucose (PDB ID: 3ZY5), was used for the modelling of the donor binding domain of fucosyltransferase 2 (FUT2). For both proteins, the coordinates were downloaded from the PDB. Residues 115–150, form the *N-Acetyllactosamine* (LacNAc) binding pocket of hemolytic lectin (LSLa), were replaced with amino residues 113–148 of fucosyltransferase 2 (FUT2). Similarly, the amino acid residues 218–248 of protein *O*-fucosyltransferase 1 (POFUT1), forming the binding pocket for the donor GDP-fucose, were replaced with the amino acid residues 174–208 of fucosyltransferase 2 (FUT2). The proteins were then prepared, optimized, and minimized by using protein preparation wizard and OPLS_2005 force field [13–15], using the Schrödinger Software Suite Maestro. These optimized and modified structures of proteins hemolytic lectin (LSLa), and protein *O*-fucosyltransferase 1 with mutated and/or replaced amino acids of fucosyltransferase 2 (FUT2) were considered to be good substitutes of the acceptor, and donor domains of fucosyltransferase 2 (FUT2), respectively [16].

### 2.3 Ligand preparation

The ligand structures were obtained from an *in*-*house* drug bank of Dr. Panjwani Center for Molecular Medicine and Drug Research (PCMD). The *LigPrep* tool was used to prepare ligands by changing their torsions, assigning them appropriate protonation states, generating stereoisomers, and determining the most probable ionization state at pH 7.0 ± 2.0 [17–19].

### 2.4 Molecular docking studies

Molecular docking studies were performed using the Glide 6.9 module of Schrödinger 2023–1. The crystal structures of the proteins 1W3F (PDB ID) and 3ZY5 (PDB ID) were downloaded from the Protein Data Bank (PDB), representing the acceptor and donor binding domains, respectively. Amino acids with similar sequences in both proteins (PDB ID: 1W3F, and 3ZY5) were mutated to match the corresponding amino acids in fucosyltransferase-2 (FUT-2), generating *in-silico* mutated crystal structures of fucosyltransferase 2 (FUT2). A grid box with dimensions of 10x10x10 Å was defined around the centroid of the co-crystallized ligands, *N*-acetyllactosamine (NLC) and GDP-fucose, in the acceptor and donor proteins, respectively. This grid box served as the ligand docking site. The Glide XP module was used to perform molecular docking, and the best-docked postures were used to interpret the final results [9, 10, 12].

### 2.5 Molecular dynamics simulations

All simulations were run on the Desmond Molecular Dynamics System, which was implemented in Maestro-Desmond Interoperability Tools (Schrödinger Release 2023–1: Desmond Molecular Dynamics System, D. E. Shaw Research, New York, 2021, and Maestro-Desmond Interoperability Tools, Schrödinger, New York, 2021.). The sequence-modified and optimized structures of mushroom lectin [PDB ID: 1W3F] and POFUT1 from *Caenorhabditis elegans* [PDB ID: 3ZY5] were used to run MD simulations. All MD simulations were run at 310 K and 1.01325 bar for 100 ns, using recording intervals of 1.2 ps for energy and 0.5 ps for trajectory. The SPC model for water was used with the OPLS3e force field [13]. The particle mesh Ewald (PME) approach, with a real space cut-off of 9.0 Å, was used to simulate electrostatic interactions. The system was linked to a 1.0-ps Nose–Hoover–Chain thermostat, and a 2.0-ps Martyna–Tobias–Klein barostat. 2.0 fs was used as the integration time step [14]. The data analysis was carried out using Desmond Simulation Event Analysis, Schrödinger Release 2023–1 [15, 20, 21].

### 2.6 MM-GBSA tool for binding energy estimation

Docking technique is used for the determination of the optimal and most appropriate orientation for molecules (protein-ligand) to interact and build a stable complex. To estimate the binding affinity, and to evaluate the docked poses, the Prime MM-GBSA (Molecular mechanics with generalised Born and surface area solvation, MM/GBSA) Schrödinger tool 2023–1 was used [19, 22]. This tool was used to re-rank the docked conformation of each listed ligand obtained by the Glide XP dock tool, as well as to estimate the relative binding affinities of these ligands [23]. A higher negative value of binding energy (measured in Kcal/mol) indicated a stronger binding affinity [24, 25].

### 2.7 STD-NMR screening experiments

All the STD-NMR experiments were performed on a Bruker AVANCE NEO 600 MHz NMR instrument at 298 K using the Stddiffesgp.3 pulse program. Protein and ligand were used at a ratio of 1:1000 folds, and 1024 scans were recorded. To saturate the protein selectively, on and off resonance irradiation was provided at -2.2 and 30 ppm, respectively. The difference spectrum was obtained by subtracting the on-resonance irradiation spectrum from the off-resonance spectrum [26, 27]. The STD amplification factor for each proton, giving the STD effect, was calculated using the formula given below:

$$STD\;Amplification\;Factor=\left(\frac{Istd}{Io}\right)\times Ligand\;Excess$$

Where *I*_*0*_ and *I*_*std*_ are signal intensities in the off and STD-difference NMR spectra, respectively.

## 3. Results and discussion

Fucosyltransferase 2 (FUT2) plays a key role in cell adhesion, cell signaling, and tumor growth. Overexpression of fucosyltransferase 2 (FUT2) has been linked to inflammation and cancer. Previous studies identified fucosyltransferase 2 (FUT2) inhibitors, although none of them have completed the clinical trial, and hence currently there is no FDA-approved drug for fucosyltransferase 2 (FUT2) inhibition [4, 28]. There are various reasons for this limited success and one of them is the lack of crystal structure of enzymes that enable us to understand the functioning of the active site [29].

During the current study, a systematic approach was used to identify new hits against fucosyltransferase 2 (FUT2) *via* computational tools (such as docking and simulation studies) and bio-physical techniques (STD-NMR) [25, 30]. Furthermore, the drug repositioning approach was used as a cost and time efficient approach in comparison to the conventional drug design strategy [28, 31].

### 3.1 Molecular docking studies of selected drugs

**Fig 1:**
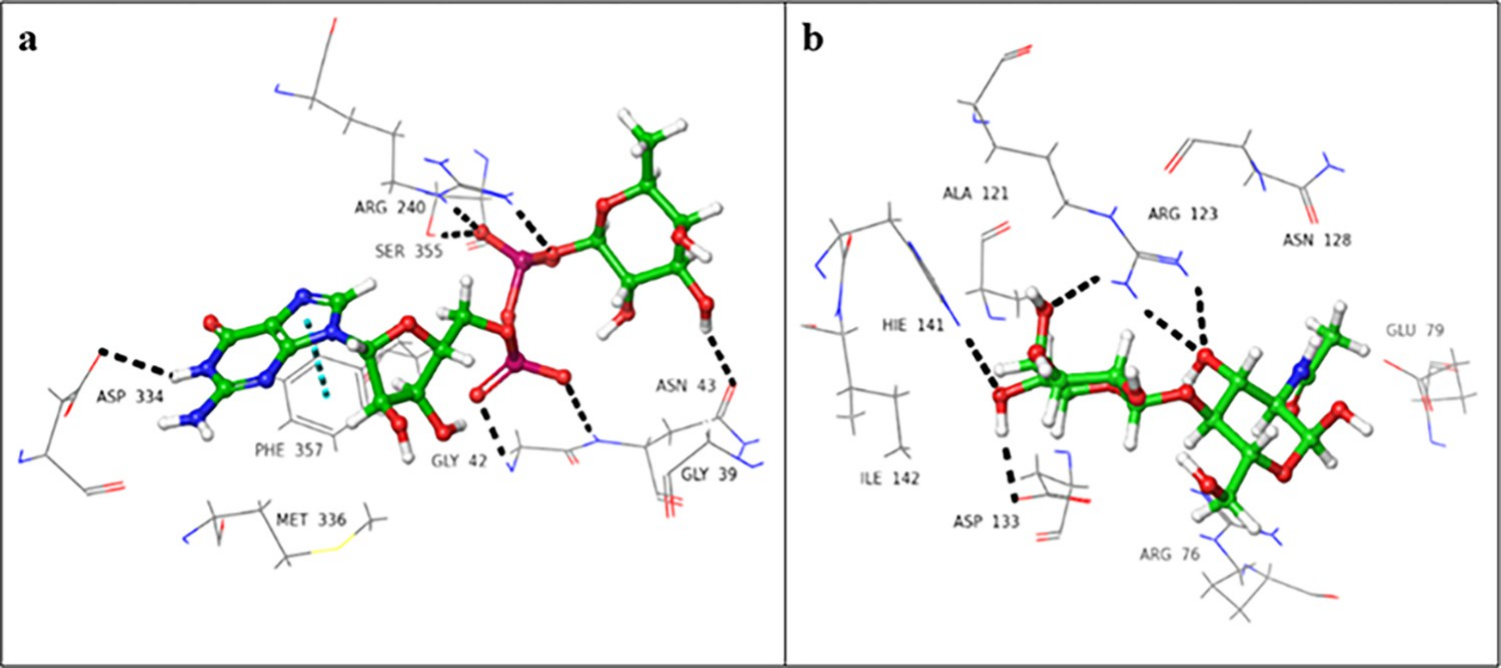
3D-Interaction diagram of (a) GDP-fucose, (b) *N*-acetyllactosamine, indicating non-covalent interactions with conserved residues of FUT2 (black dotted lines: hydrogen bonds, cyan dotted lines: π-π stacking interactions).

As the 3D structure of fucosyltransferase 2 (FUT2) is still to be elucidated, we used the *in silico* prediction of binding sites for the donor and acceptor domains, individually. We have previously reported the homology model for both domains of fucosyltransferase 2 (FUT2) [26]. The important amino acid residues that were involved in the binding of the donor and acceptor substrates includes Arg240, Ser355, Phe357, Asp334 (donor binding site), and Arg123, Asp133, and His141 (acceptor binding site) (**Fig 1**).

Around 500 US FDA-approved drugs, available in the drug bank of Dr. Panjwani Center for Molecular Medicine and Drug Research (PCMD), were selected for the study. These drugs were screened using *in-silico* docking studies against the homology model of fucosyltransferase 2 (FUT2). Among them, the drugs that showed higher docking scores (**S2 Table**), and interactions with key enzyme residues were selected for ligand-receptor interactions study *via* STD-NMR spectroscopy. This approach identified five drugs that exhibited substantial STD effects: enalaprilat, ibuprofen, ascorbic acid, acarbose, and ceftriaxone sodium. The current therapeutic use of these drugs is presented in **S1 Table**.

All the five drugs showed interactions with the catalytically important residues of donor and acceptor binding domains in *in-silico* studies. However, the docking scores were more significant for the donor binding domains, in comparison to the acceptor binding domains. These results were consistent with the literature reported about fucosyltransferases (FUTs), as they have a deep binding site for the donor substrate, while the acceptor binding site is shallow. These drugs mainly interacted *via* hydrogen bonding, and in some cases salt bridge formation, as well as *π*-cationic and *π*-*π* stacking interactions.

**Fig 2:**
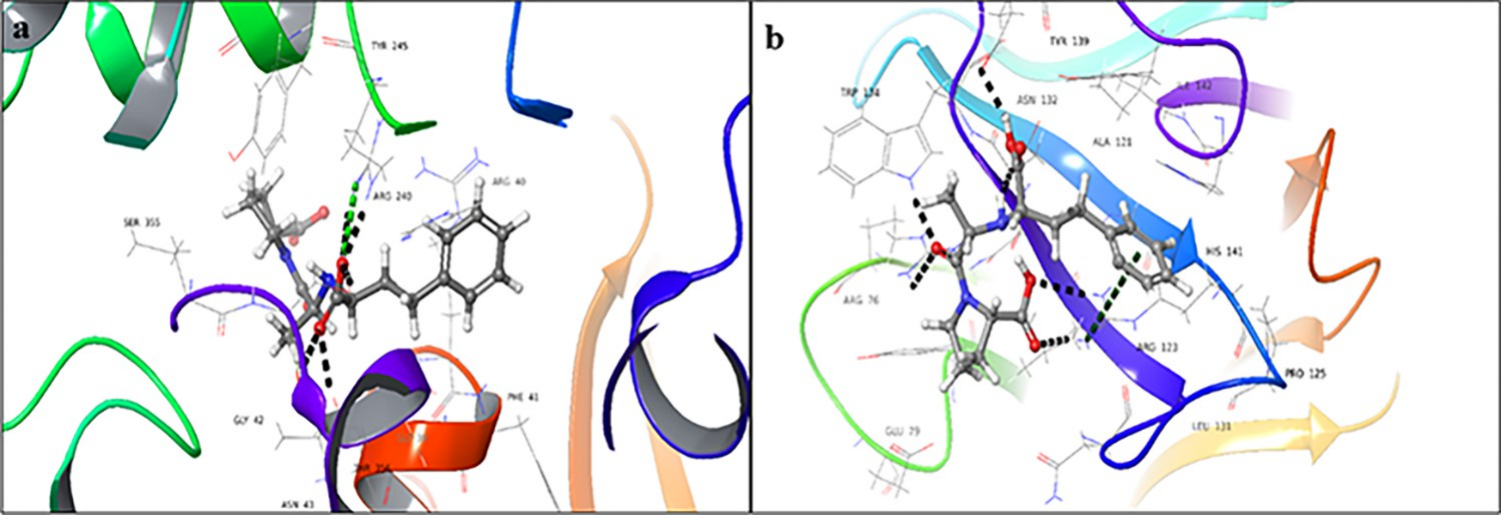
3D-Docked poses of enalaprilat dehydrate **(1)** in the (**a**) donor, and (**b**) acceptor domains of FUT2 (Black dotted lines: hydrogen bonds, cyan dotted lines: π-π stacking interactions).

Enalaprilat dehydrate **(1)** showed the highest docking score for the donor binding domain (-9.474), in comparison to the acceptor binding domain (-5.363). The drug showed interactions mainly *via* the aliphatic chain, instead of the aromatic ring in the case of donor binding domains (Fig 2A). The carboxylic group was involved in hydrogen bonding with Gly42, Thr356, and Arg240. A salt bridge formation was also observed with Arg240. For the acceptor binding domain, it interacted *via* aliphatic as well as aromatic chains with Arg76, Trp134, and Arg123 *via* hydrogen and π-cationic interactions, respectively (Fig 2B). The phenyl ring of the drug interacted with Arg123 *via* π-cationic interactions. The binding affinity of enalaprilat dehydrate (**1**) for the donor binding domain was found to be -96.46 Kcal/mol, indicating a highly stable protein-ligand complex, whereas the acceptor domain exhibited a binding affinity of -29.04 Kcal/mol. These results reveal that the protein-ligand complex, formed with the donor binding domain, is significantly more stable than that formed with the acceptor binding domain.

**Fig 3:**
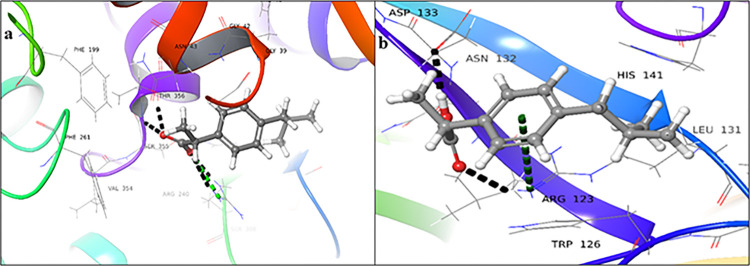
3D-Docked poses of ibuprofen **(2)** in the (a) donor, and (b) acceptor domains of FUT2 (Black dotted lines: hydrogen bonds, dark green dotted lines: π cationic interactions, light green: salt bridge).

Ibquprofen (**2**) showed a significant docking score for the donor binding domain (-7.077), while a low docking score for the acceptor binding domain (-2.583). Carboxyl moiety of the drug showed hydrogen bonding, with Arg240, Ser356, and Thr356 in the donor binding domain (Fig 3A). While, in the acceptor binding domain, the drug showed hydrogen bonding, with Arg76, Asp133, and Arg123. The Phenyl ring of the ligand interacts with the NH_2_ group of Arg123 *via* a π-cation bond (Fig 3B). The docking results for ibuprofen (**2**) revealed a binding energy of -63.02 Kcal/mol for the donor binding site, indicating a strong ligand-receptor interaction, whereas the acceptor binding site showed a binding energy of -32.09 Kcal/mol, suggesting a relatively weaker interaction. These findings suggest that the protein-ligand complex formed at the donor active site is more stable than that formed at the acceptor active site.

**Fig 4:**
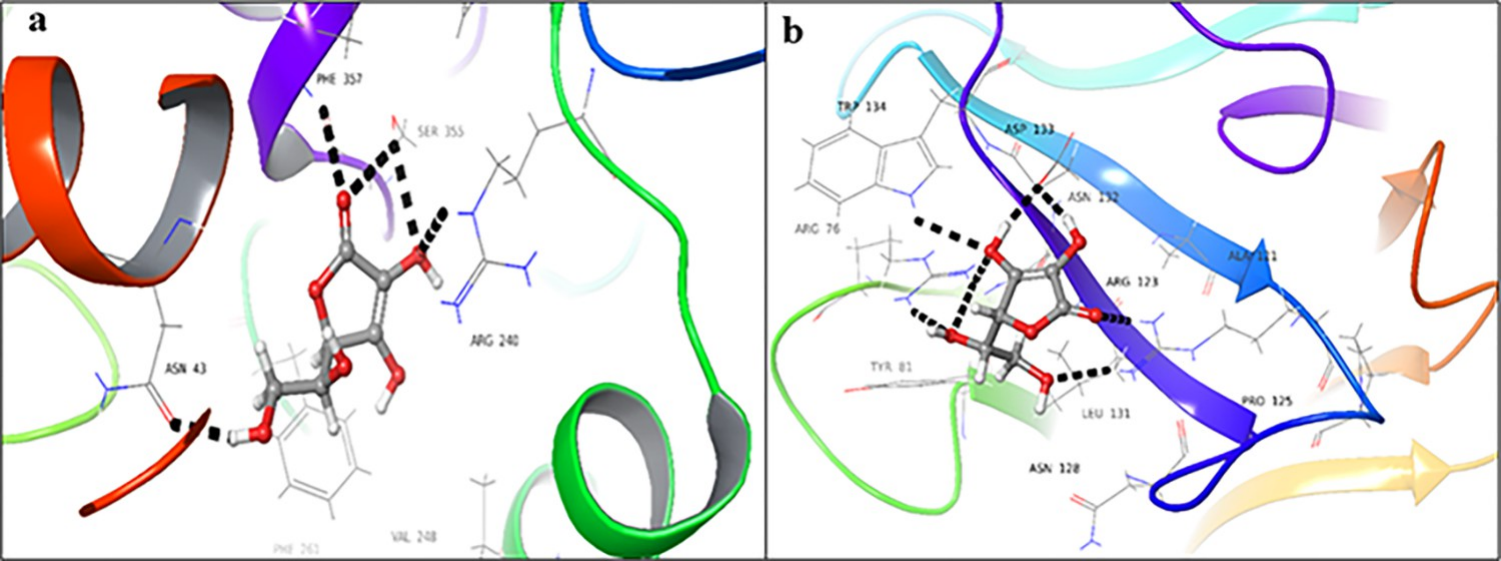
3D-Docked poses of ascorbic acid **(3)** in the (**a**) donor, and (**b**) acceptor domains of FUT2 (Black dotted lines: hydrogen bonds, light green: salt bridge).

Ascorbic acid (**3**) showed a significant docking score with donor binding domain (-9.149), with a comparatively a lower docking score with acceptor binding domain (-4.425). It interacts *via* hydrogen bonding and salt bridge formation with the donor (Asn43, Arg240, Ser355, and Phe357) (Fig 4A), as well as the acceptor active sites (Arg123, Asp133, Glu137, and His141) (Fig 4B). Using the MMGBSA tool, we estimated the stability of the protein-ligand complex and found that the donor binding domain exhibited a binding energy of -60.23 Kcal/mol, indicating high stability, whereas the acceptor binding domain showed a binding energy of -21.12 Kcal/mol, suggesting relatively lower stability. These results indicate that the protein-ligand complex, formed at the donor ligand-receptor domain, is more stable than that formed at the acceptor domain of the protein.

**Fig 5:**
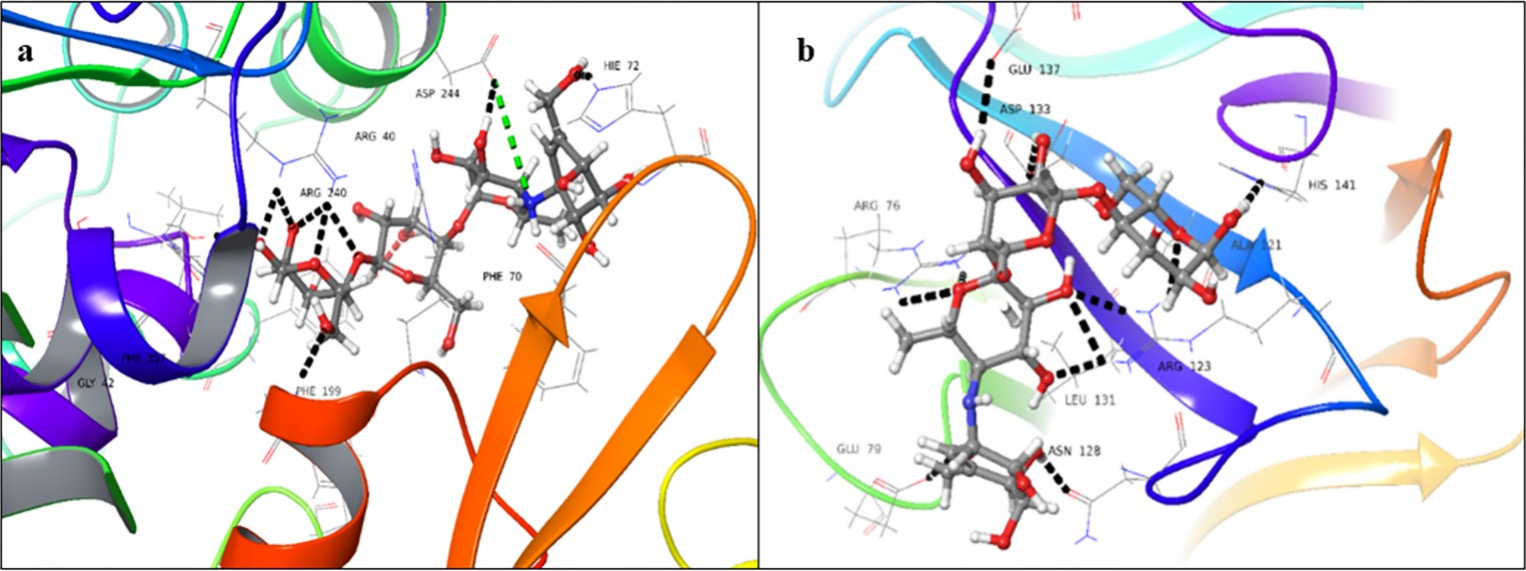
3D-Docked poses of acarbose (**4**) in the (**a**) donor, and (**b**) acceptor domains of FUT2 (Black dotted lines: hydrogen bonds, light green: salt bridge).

Acarbose (**4**) showed significant docking scores for the donor (-11.013), as well as for acceptor binding domains (-9.362). It showed H-bonding interactions with Arg40, Asp309, Arg330, and Asp334, with the donor binding sites and a salt bridge formation with Asp244 (Fig 5A). While in the acceptor binding site, it showed hydrogen bonding with ligand through Arg76, Glu79, Arg123, Trp126, Asp133, Glu137, Tyr139, and Ile142 (Fig 5B). The donor domain of the protein exhibited

A significantly higher binding energy of -76.06 Kcal/mol as compared to the acceptor domain, which showed a binding energy of -66.95 Kcal/mol, indicating that the donor domain forms a more stable complex with the ligand.

**Fig 6:**
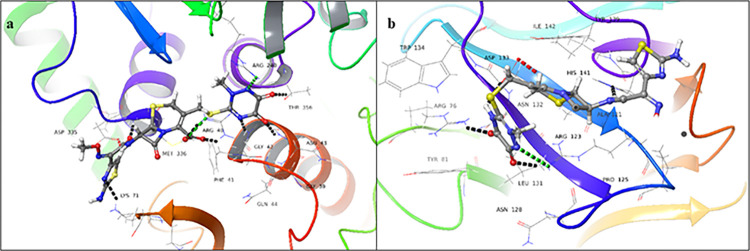
3D-Docked poses of ceftriaxone sodium **(5)** in the (**a**) donor, and (**b**) acceptor domains of FUT2 (Black dotted lines: hydrogen bonds, light green: salt bridge, red: aromatic hydrogen bonds).

Ceftriaxone sodium (**5**) showed a significant docking score for the donor (-8.241), as well as for acceptor binding domains (-4.002). It showed hydrogen bonding with Met336, Arg40, Asn43, Thr356, and Phe357 in the donor binding domain (Fig 6A). While in the acceptor binding site, it showed hydrogen bonds with Arg76, Arg123, Trp126, Asp133, and His141 (Fig 6B). Ceftriaxone sodium (**5**) exhibited a strong binding affinity with the donor domain, with a binding energy of -79.89 Kcal/mol, whereas the acceptor domain showed a significantly weaker binding energy of -38.26 Kcal/mol. These results indicated that the protein-ligand complex formed with the donor domain is substantially more stable than that formed with the acceptor domain.

### 3.2 Epitope mapping *via* STD-NMR

The STD-NMR technique was used to validate the ligand receptor interactions, predicted through docking studies. STD-NMR is among the most powerful biophysical techniques to study ligand-receptor interactions [32]. The experiment is based on the radio frequency saturation transfer from the receptor molecule to the bound ligand. The intensities of the ligand protons in the 1D STD-spectrum reflects their contact with the protein surface; stronger STD indicates shorter distance between ligand protons and receptor molecules, and *vice versa* [33]. This allows the epitope maps of the bound ligand *via* analyzing the degree of saturation of its individual protons. The relative STD percentage of each individual ligand proton is analyzed by arbitrarily assigning 100% saturation to the most intense signal. Thus, the epitope mapping identifies the moieties of the ligand that are involved in molecular recognition of the binding site of the receptor [34].

#### Enalaprilat dihydrate (1)

**Fig 7:**
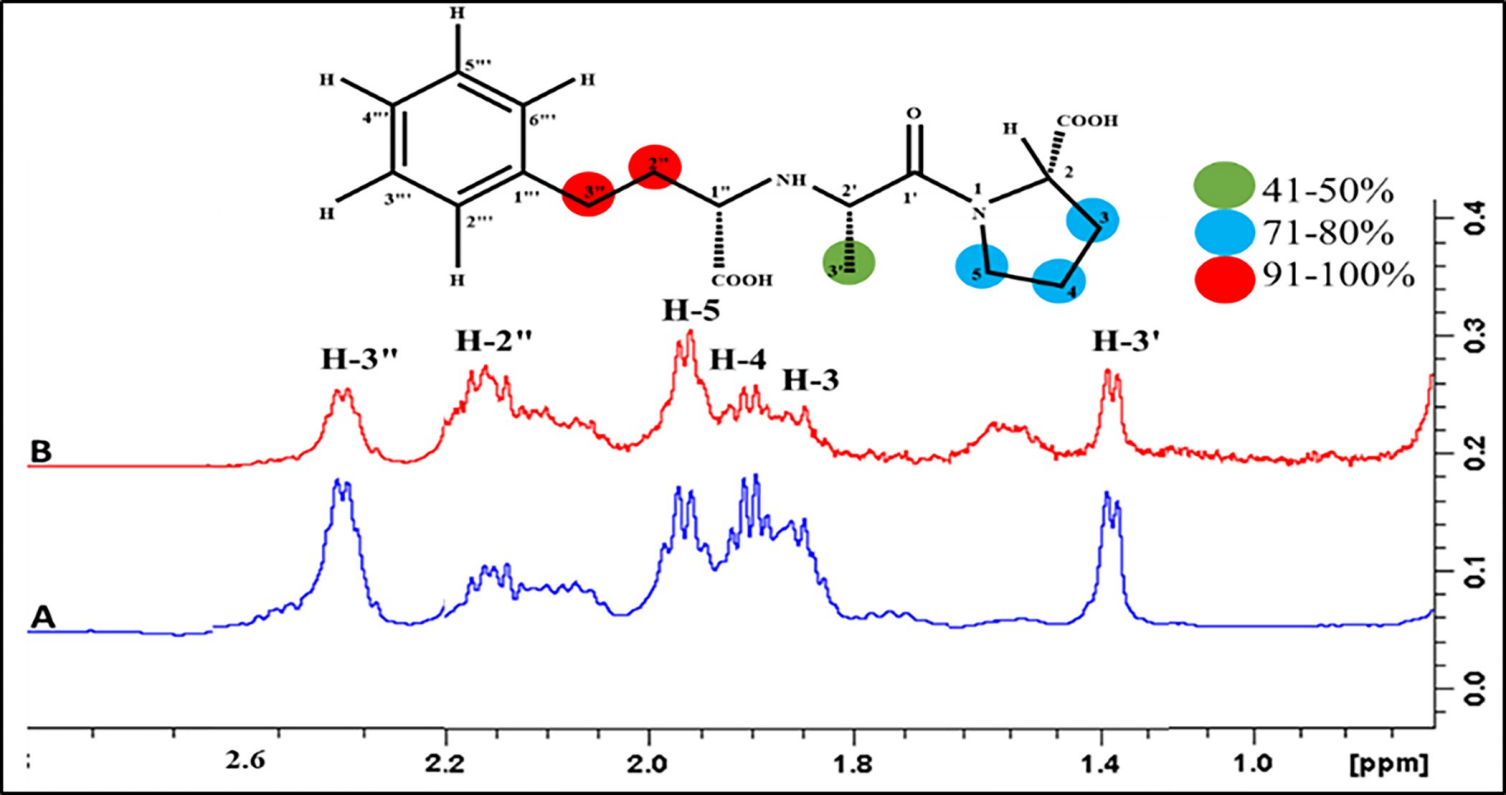
(A) Reference ^1^H-NMR spectrum of enalaprilat dehydrate **(1)**. (B) Difference spectrum (STD-NMR) of enalaprilat dihydrate with FUT2.

The aromatic and aliphatic protons in enalaprilat dehydrate (**1**) showed interactions with the fucosyltransferase 2 (FUT2) enzyme (Fig 7). The aliphatic protons at C-2" and C-3" showed the highest STD-effects and were assigned to receive 100% saturation transfer. The pentose sugar of enalaprilat dehydrate (**1**), also played a role in the interactions with the receptor protein. For instance, H-4 and H-5 exhibited 77%, while H-2 received 73% saturation transfer. The C-3’ proton received 46% saturation transfer. The group epitope mapping (GEM) analysis indicated that the aliphatic moiety of the drug was in close proximity to the receptor protein fucosyltransferase 2 (FUT2). These results were in agreement with the docking studies, where this drug interacted mainly *via* aliphatic protons in the case of the donor binding domain.

#### Ibuprofen (2)

**Fig 8:**
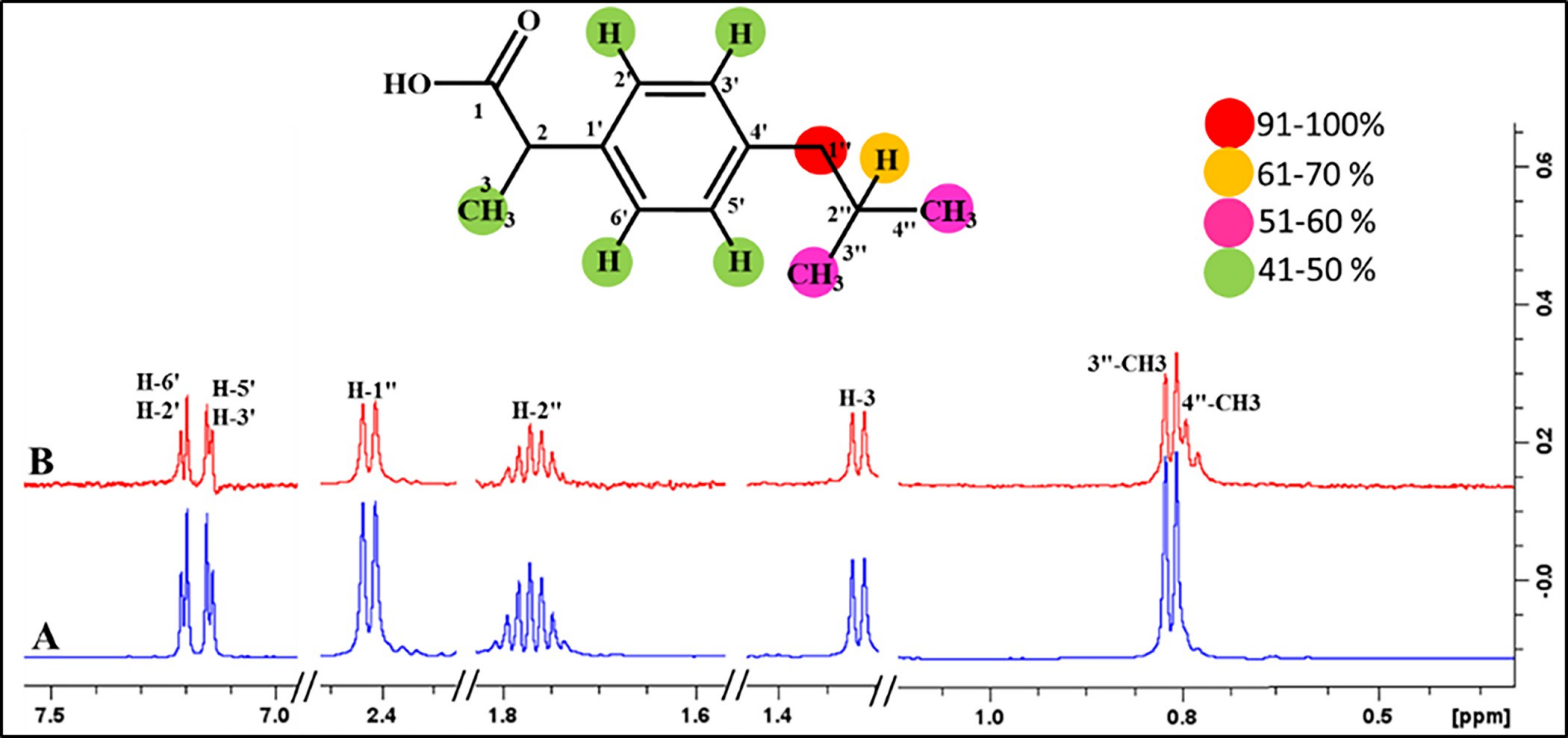
(A) Reference ^1^H-NMR spectrum of ibuprofen **(2)**. (B) Difference spectrum (STD-NMR) of ibuprofen with FUT2. (*) = Solvent peak.

The STD-NMR spectrum of ibuprofen **(2)** displayed multiple binding networks (Fig 8). H-1" displayed 100% saturation transfer, which showed a high intensity signal, as compared to other protons, followed by H-2’, which showed 64% saturation transfer. The C-3’’ and C-4’’ showed 54% saturation transfer. Similarly, aromatic H-2’/H-6’, and H-3’/H-5’ showed 50% saturation transfer. The C-3 received 46% saturation transfer. Comparatively, H-1", and H-2" displayed the highest intensity signals in the STD-difference spectrum, which indicated their close contacts with the receptor protein. However, in the case of docking studies, the carboxylic group showed a major interaction.

#### Ascorbic acid (3)

**Fig 9:**
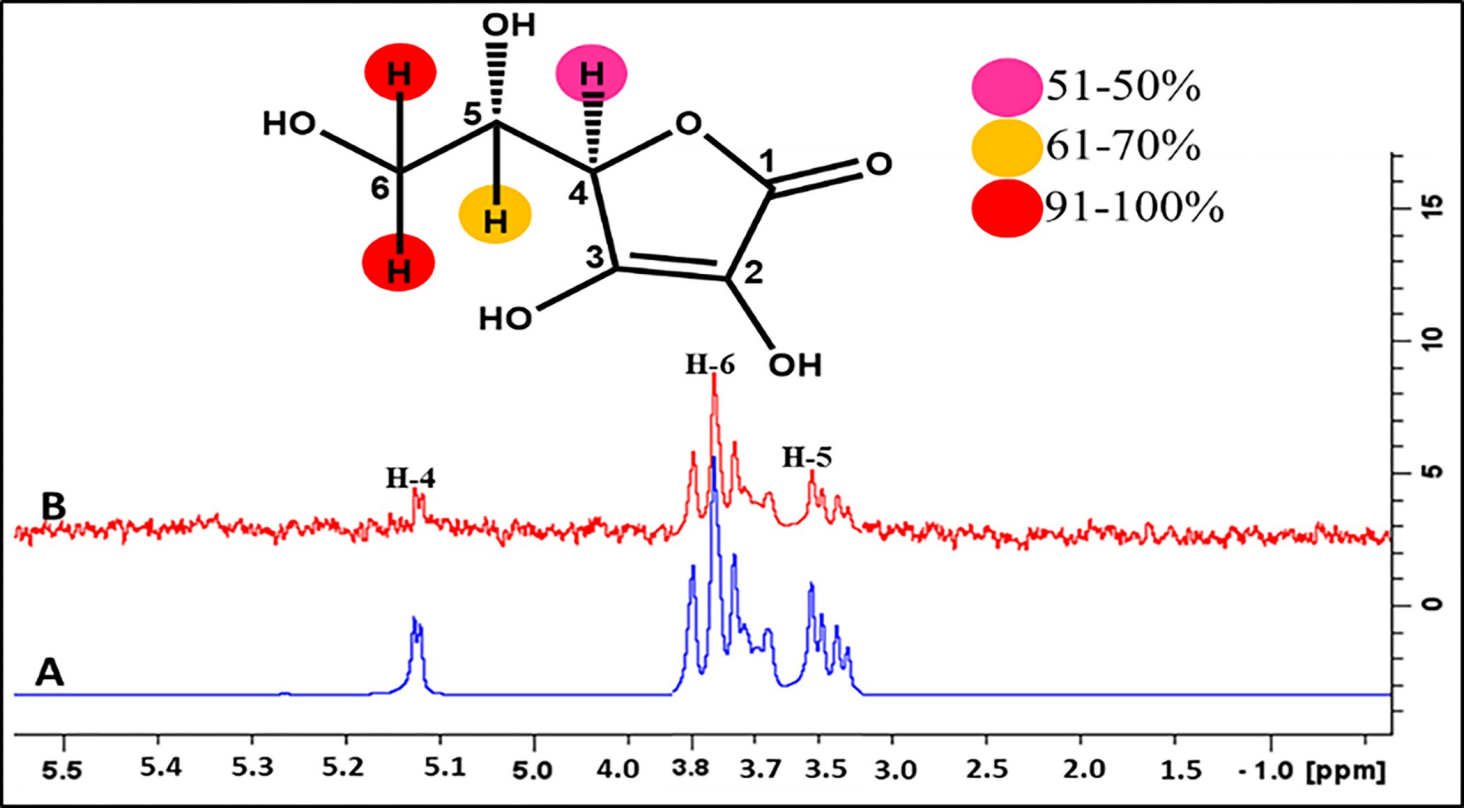
(A) Reference ^1^H-NMR spectrum of ascorbic acid **(3)**. (B) Difference spectrum (STD-NMR) of ascorbic acid **(3)** with FUT2.

Ascorbic acid (**3**) has a single aromatic ring along with the aliphatic chain (Fig 9). The STD-NMR spectrum showed that H-6 showed 100%, whereas the H-5 and H-4 received 82% and 60% saturation transfer, respectively. Docking studies predicted the interactions of the whole ligand molecule with the recptor protein as it is a small compound. In STD-NMR, we were unable to analyze the hydroxyl protons of the ring.

#### Acarbose (4)

**Fig 10:**
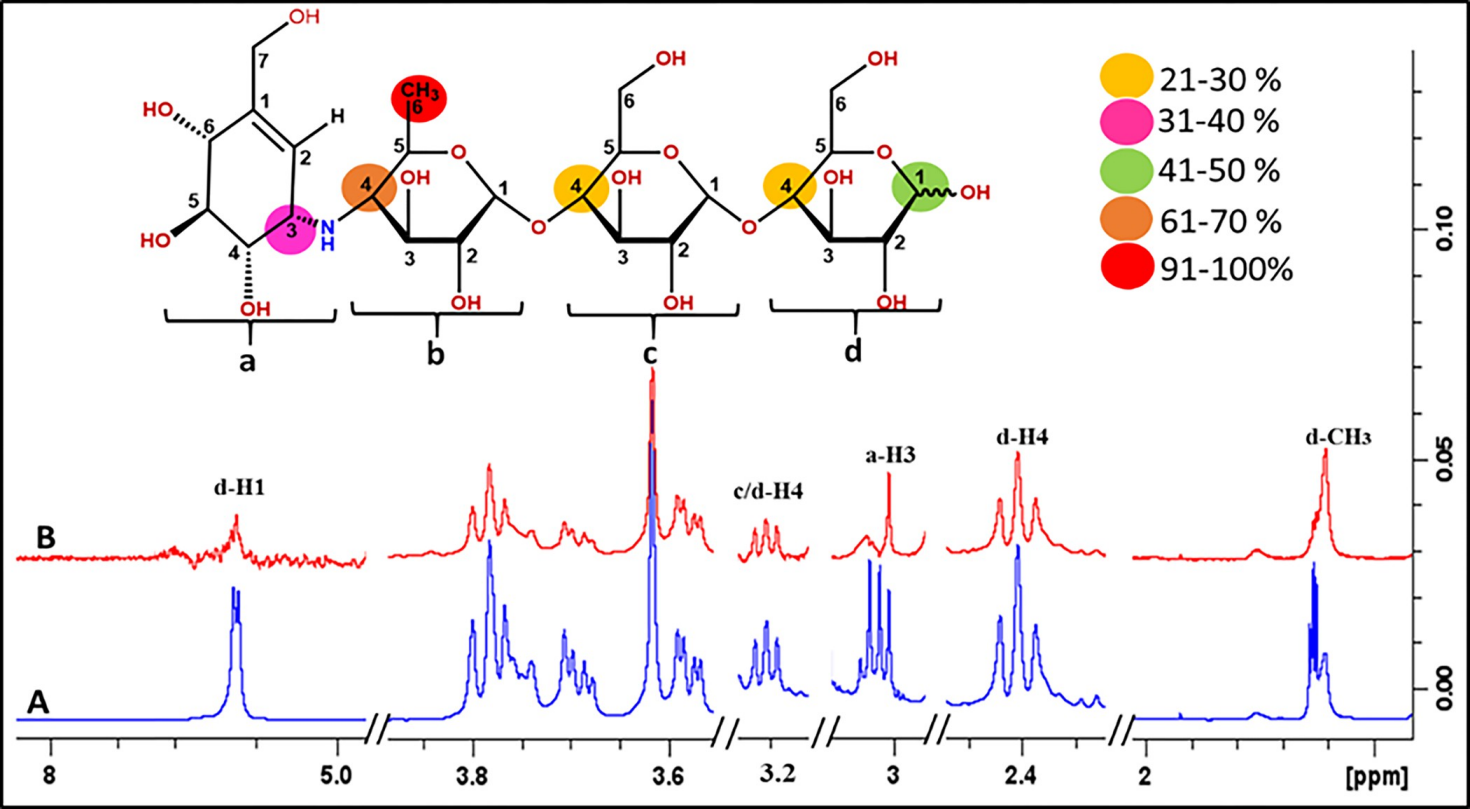
**(A)** Reference ^1^H-NMR spectrum of acarbose **(4)**. **(B)** Difference spectrum (STD-NMR) of acarbose. with FUT2.

Acarbose (**4**) consists of sugar rings involved in multiple interactions with the receptor protein fucosyltransferase 2 (FUT2) (Fig 10). For instance, the methyl protons in C-6 in ring (b) displayed 100% saturation transfer, and indicated close contact with the receptor protein, whereas H-4 (in ring b) showed 68% saturation transfer. Similarly, 36% saturation transfer from the receptor protein was observed for H-3 of the ring (a). Protons in rings (c) and (d) in C-4 exhibited ligand interactions with receptor proteins, each of which showed 28% saturation transfer. H-1 of the ring (d) showed 44% saturation transfer. For the few protons of the sugar ring, epitope mapping and relative percentages calculations were not possible, as the signals of these protons were overlapped. The results were in agreement with docking studies that predicted the interaction of various groups of rings a-d.

#### Ceftriaxone sodium (5)

**Fig 11:**
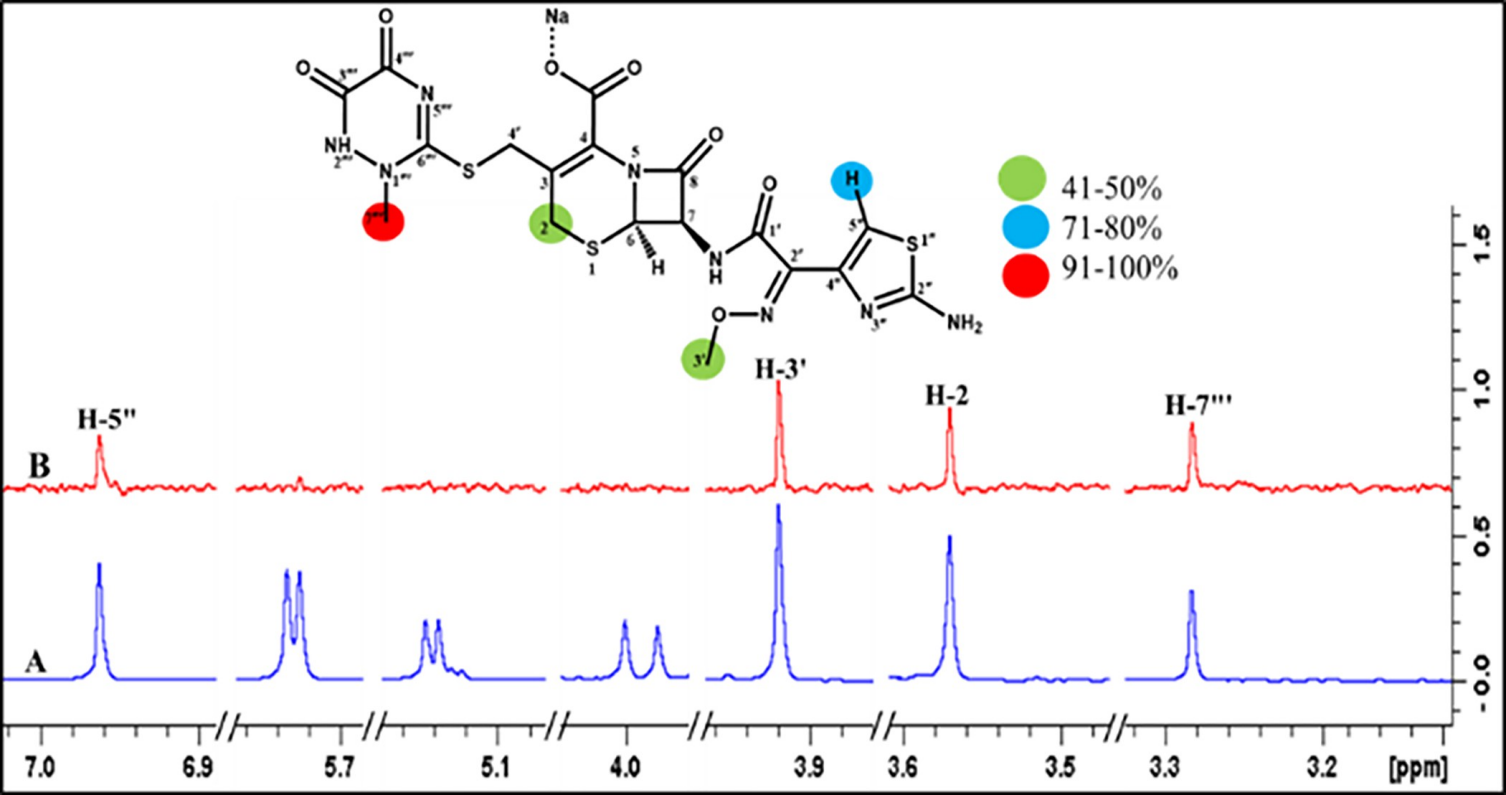
**(A)** Reference ^1^H-NMR spectrum of ceftriaxone sodium **(5)**. **(B)** Difference spectrum (STD-NMR) of ceftriaxone sodium with FUT2.

Ceftriaxone sodium (**5**) showed a number of interactions with the receptor protein (Fig 11). For instance, the C-7‴ protons demonstrated 100% saturation transfer, indicating the close contact of the traizine ring with the receptor protein. In the thaizole ring, H-5’’ received 74% saturation from the receptor protein. While H-2 and the methyl group at C-3’ exhibited 42% saturation transfer. Docking studies also showed interactions of the triazine ring, mainly with the protein followed by other parts of the drug.

### 3.3 Molecular dynamics simulations

As fucosyltransferases (FUTs) are bi-substrate enzymes with donor and acceptor binding sites, MD simulations were performed to predict the binding site interactions for each drug. STD-NMR is a ligand based technique, and one of the limitations is that it does not provide any information about protein structure and conformation in the context of its binding related with the ligand. Docking studies on the other hand, provide ligand-receptor interactions at atomic level, but the receptor is in rigid conformation. Hence, to overcome these limitations, MD simulations are the best choice to gain insights about the ligand-receptor complex in a dynamic environment [20, 29]. In order to understand the dynamics of ligand binding with the active site of the enzyme, the molecular dynamics simulation tool examines the stability and dynamic fluctuations of the ligand-protein complex in a simulated biological environment. Hence, an MD simulation for 100 nsec was run for the five related drugs to analyze the drug-enzyme complex stability. The results were interpreted *via* analyzing RMSD, RMSF, and non-covalant interactions of the drug with the enzyme [35, 36].

#### Enalaprilat dihydrate (1)

**Fig 12:**
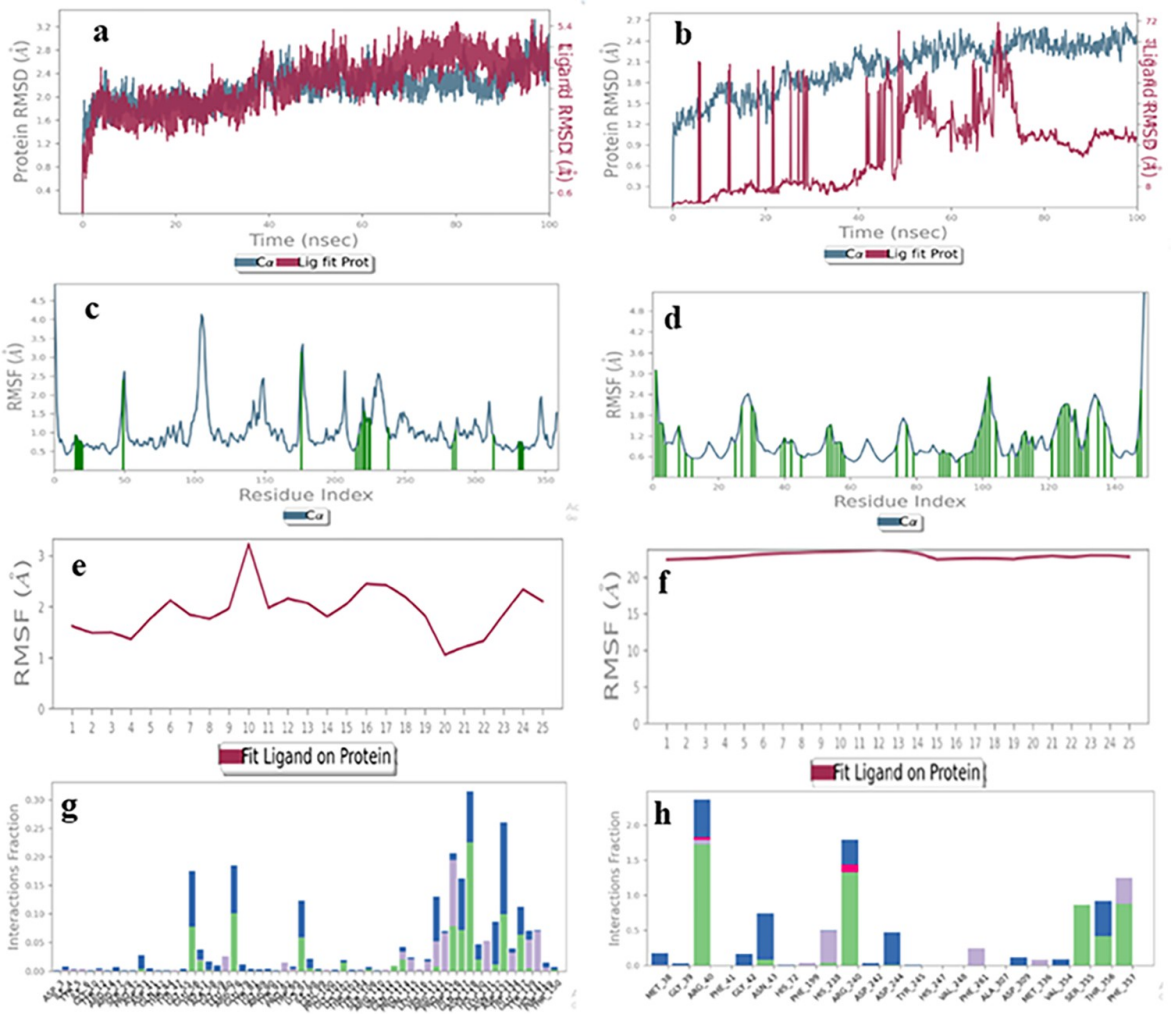
The molecular dynamics trajectories of enalaprilat dihydrate with proteins acceptor (**a**, **c**, **e**, **g**) and donor binding sites (**b**, **d**, **f**, **h**). **(a, b)** Protein-ligand RMSD **(c, d)** Protein RMSF (**e**, **f**) Ligand RMSF, **(g, h)** Protein-ligand interaction histogram.

MD Simulation of enalaprilat dehydrate (**1**) with acceptor and donor binding domains indicated the probability of its binding in the donor active site. The RMSD plot of the ligand-receptor complex showed 1–1.2 Å for the donor binding domain (Fig 12A), while for the acceptor, the ligand RMSD was more than 40Å, indicating that the ligand might get diffused away from the acceptor binding site (Fig 12B). The RMSF of the protein showed acceptable value *i*.*e*., less than 2Å for interacting residues in donor, as well as acceptor active sites (Fig 12C and 12D). However, the ligand RMSF indicated that the drug was more stable in the donor binding domain (RMSF value less than 2.5 Å), while in the case of an acceptor it might get dissociated as RMSF was above 20 Å (Fig 12E and 12F). The histogram exhibited the active amino acids that interacted with ligand in the binding pocket, such as, Arg40, Asn43, His238, Arg240, Asp244, Ser355, Thr356, and Phe357 *via* hydrogen bonding, water bridge formation, and hydrophobic contacts for more than 50% of the simulation time with enalaprilat dihydrate, indicating ligand stability in the donor binding site (Fig 12G), While, in case of acceptor active site, none of the interaction between drug and enzyme was stable for more than 30% of the simulation time (Fig 12H). Hence, it was concluded that enalaprilat dehydrate (**1**) is a better binder for donor active site [37].

#### Ibuprofen (2)

**Fig 13:**
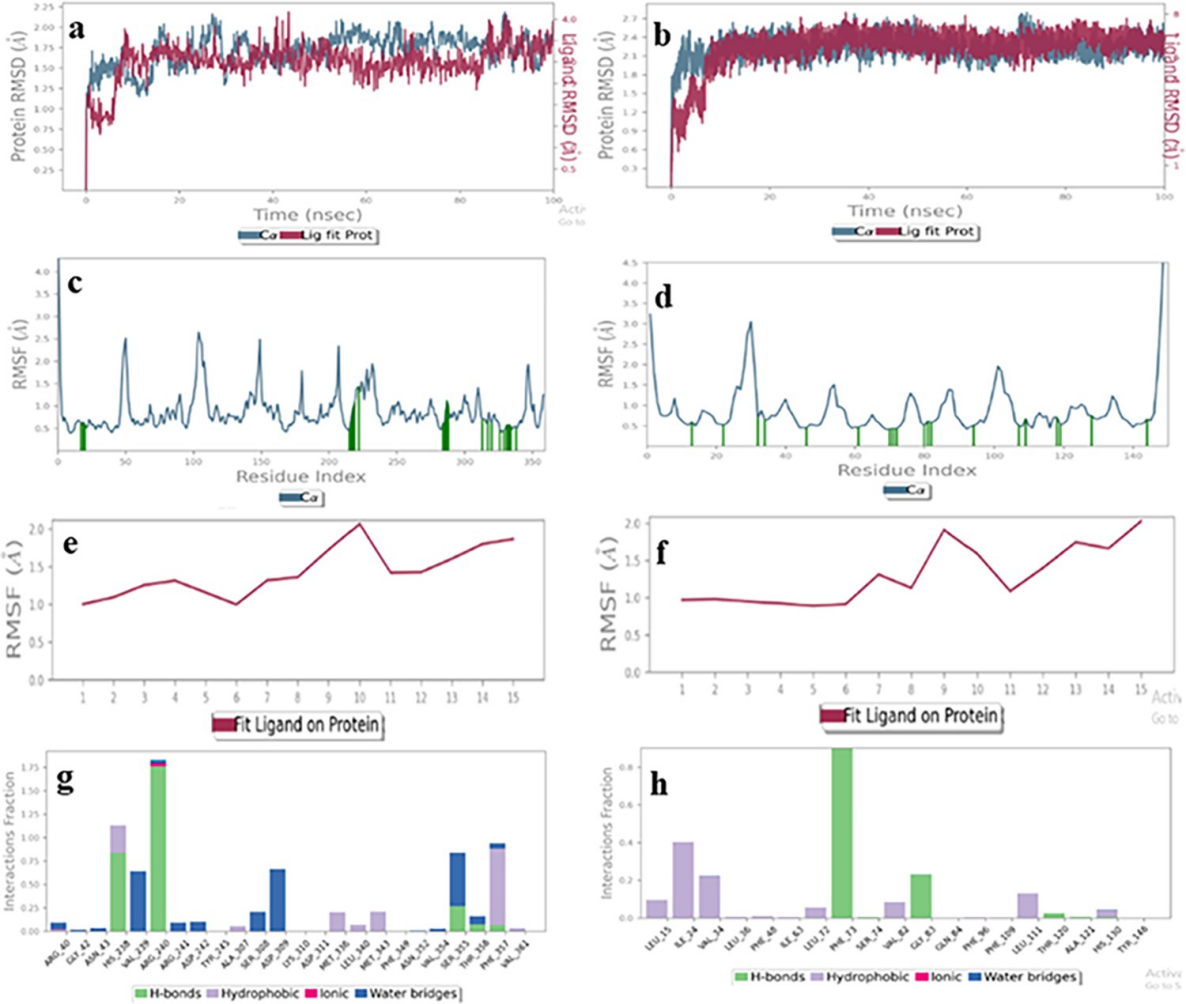
The molecular dynamics trajectories of ibuprofen **(2)** with protein acceptor (**a**, **c**, **e**, **g**) and donor binding sites (**b**, **d**, **f**, **h**). **(a, b)** Protein-ligand RMSD **(c, d)** Protein RMSF (**e**, **f**) Ligand RMSF, **(g, h)** Protein-ligand interactions histogram.

MD Simulation of ibuprofen (**2**) showed equal probability of binding at the donor and acceptor binding sites. The RMSD plot of the ligand-receptor complex showed perturbations in the acceptable range *i*.*e*., less than 1 Å for both binding sites (Fig 13A and 13B). Similarly, the RMSF of the protein (Fig 13C and 13D) and ligand were also less than 2 Å for both the binding sites (Fig 13E and 13F). Hence, we concluded that ibuprofen (2) has an equal probability of binding of the acceptor and donor binding sites. However, the interactions of proteins with the ligand were more in the case of the donor binding domain. For instance, His238, Val239, Arg240, Asp309, Ser355, and Phe357 interacted *via* hydrogen bonding and hydrophobic contacts for more than 50% of the simulation time (Fig 13G and 13H), while there was one hydrogen bond with Phe73 that retained for more than 50% of the simulation time. This indicated that ibuprofen (**2**) can better be accommodated in the donor binding site [38].

#### Ascorbic acid (3)

**Fig 14:**
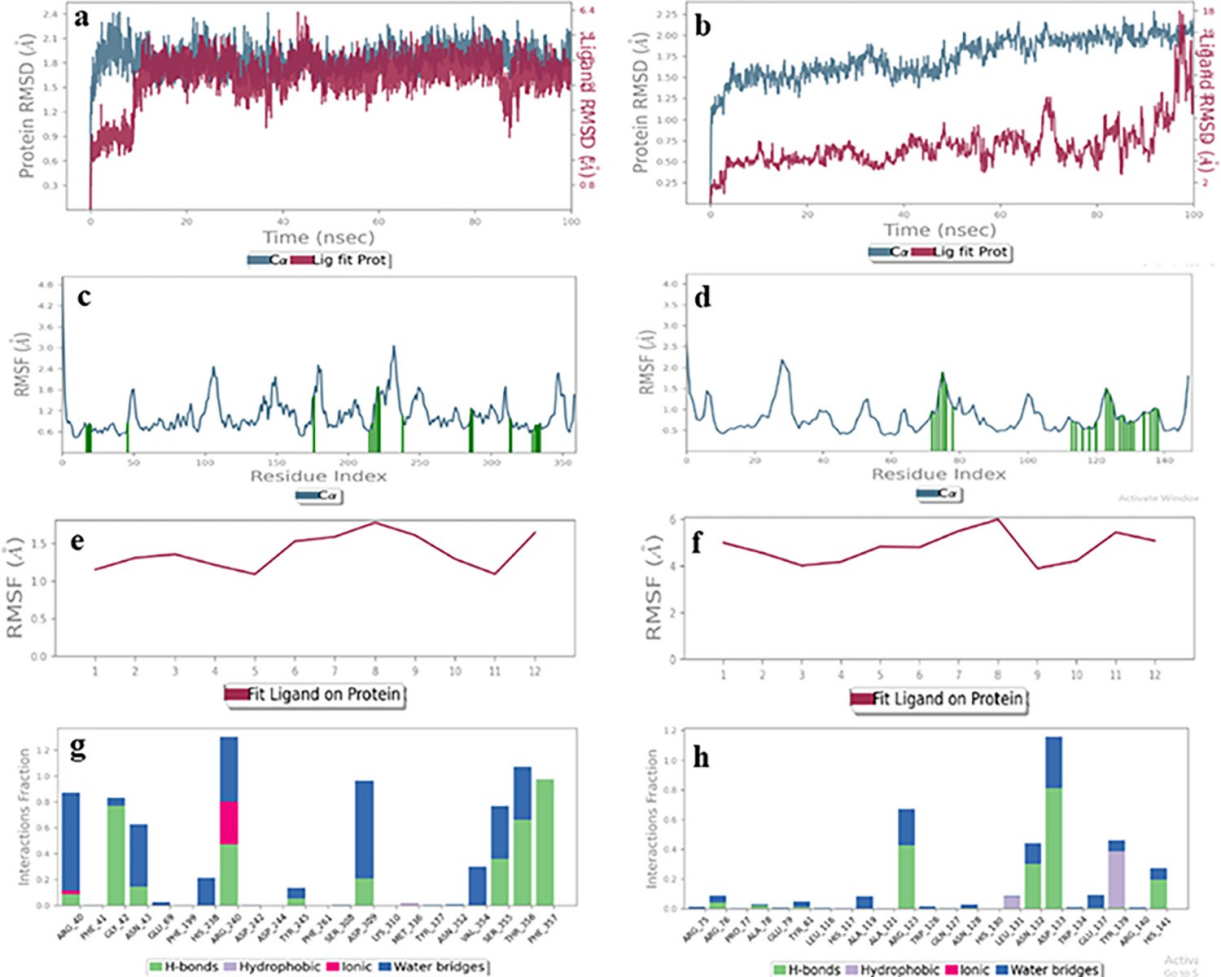
The molecular dynamics trajectories of ascorbic acid with protein acceptor (**a**, **c**, **e**, **g**) and donor binding sites (**b**, **d**, **f**, **h**). **(a, b)** Protein-ligand RMSD **(c, d)** Protein RMSF (**e**, **f**) Ligand RMSF **(g, h)** Protein-ligand interaction histogram.

The RMSD plot of the ligand-receptor complex for ascorbic acid (**3**) showed perturbations in the acceptable range i.e., less than 3 Å for both binding sites (Fig 14A and 14B). Similarly, the RMSF of the protein was less than 3 Å for the donor, as well as the acceptor binding domains (Fig 14C and 14D). However, the ligand RMSF showed that the ligand was more stable in the donor binding site (RMSF less than 2 Å), while it fluctuated in the acceptor binding site (RMSF more than 4 Å) (Fig 14E and 14F). Hence, we concluded that acorbic acid can best accomodate in the donor’s bining pocket. Interestingly, the interactions of proteins with the ligand were more in the case of the donor binding domain. For instance, Arg40, Gly42, Asn43, Arg240, Asp309, Ser355, Thr356, and Phe357 residues interacted *via* hydrogen bonding, and hydrophobic contacts and few ioinc interactions, for more than 50% of the simulation time, indicating ligand stability in the donor binding site (Fig 14G and 14H). While, none of the interaction was retained for more than 50% of the simulation time in the case of the acceptor binding domain, indicating ascorbic acid is a better binder for the donor binding site of fucosyltransferase 2 (FUT2) [39].

#### Acarbose (4)

**Fig 15:**
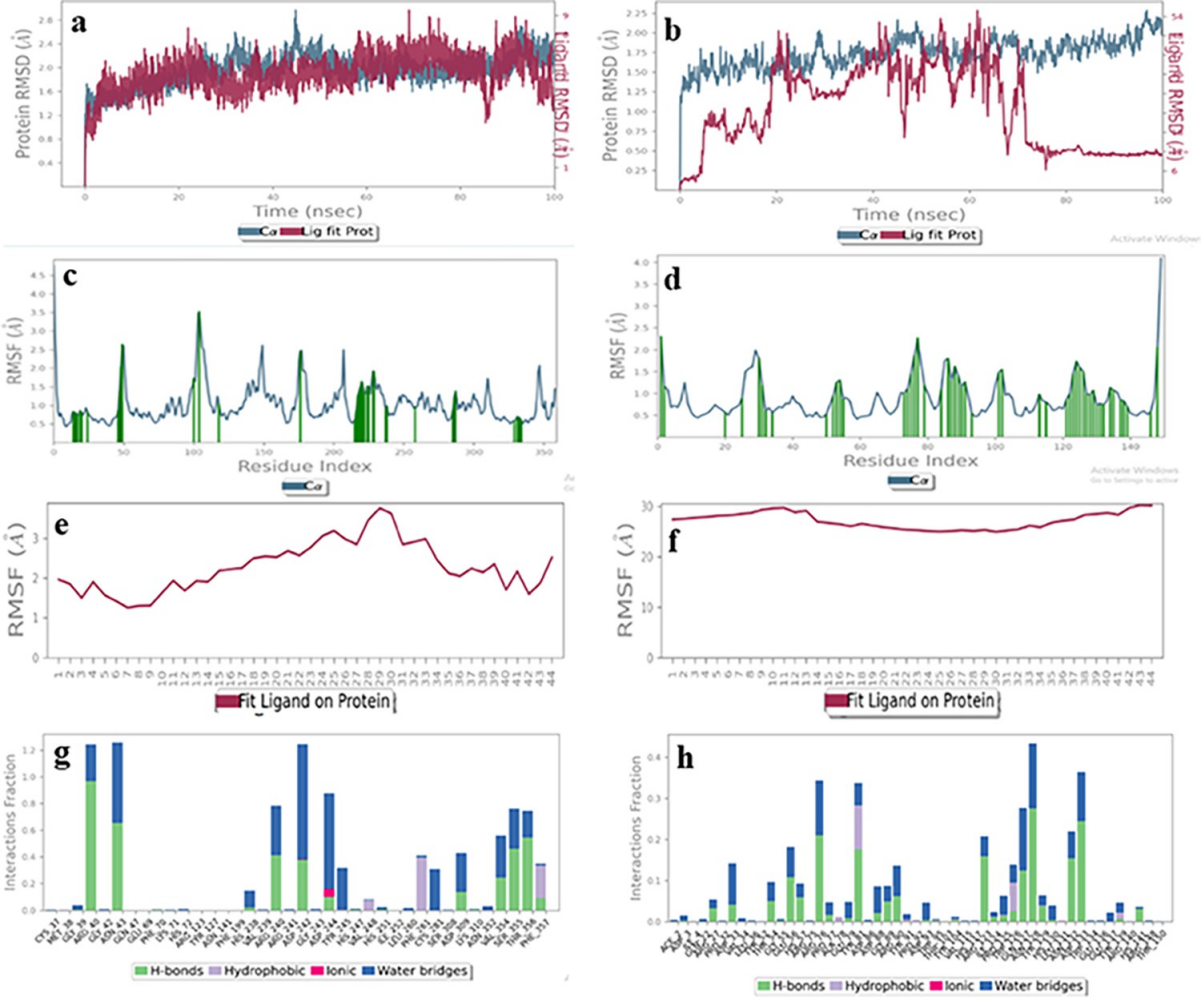
The molecular dynamics trajectories of acarbose **(4)** with protein acceptor (**a**, **c**, **e**, **g**) and donor binding sites (**b**, **d**, **f**, **h**). **(a, b)** Protein-ligand RMSD **(c, d)** Protein RMSF (**e**, **f**) Ligand RMSF, **(g, h)** Protein-ligand interaction histogram.

The RMSD plot of the ligand-receptor complex for acarbose (**4**) showed less than 3 Å perturbation in protein structure for both the binding sites. However, the ligand RMSD showed that it was more stable in the donor binding site (RMSD less than 3 Å), while it fluctuated more in the acceptor binding site (RMSD more than 20 Å) (Fig 15A and 15B). Similarly, the RMSF of protein was similar for both the binding sites (less than 3.5 Å) (Fig 15C and 15D). Interestingly, the ligand RMSF showed better binding in the donor binding domain (less than 3.5 Å), while it gets dissciated from the acceptor binding site (more than 30 Å) (Fig 15E and 15F). The interactions of protein with the ligand were also more in the case of the donor binding domain. For instance, Arg40, Asn43, Arg240, Asp242, Asp244, Asp309, Val354, Ser355, and Thr356 residues interacted via hydrogen bonding and water bridge formation interactions were retained for more than 50% of the simulation time (Fig 15G). While none of the interaction was retained for more than 50% of the simulation time (Fig 15H), in the acceptor binding domain, indicating acarbose, is a better binder for the binding site of fucosyltransferase 2 (FUT2) [40].

#### Ceftriaxone sodium (5)

**Fig 16:**
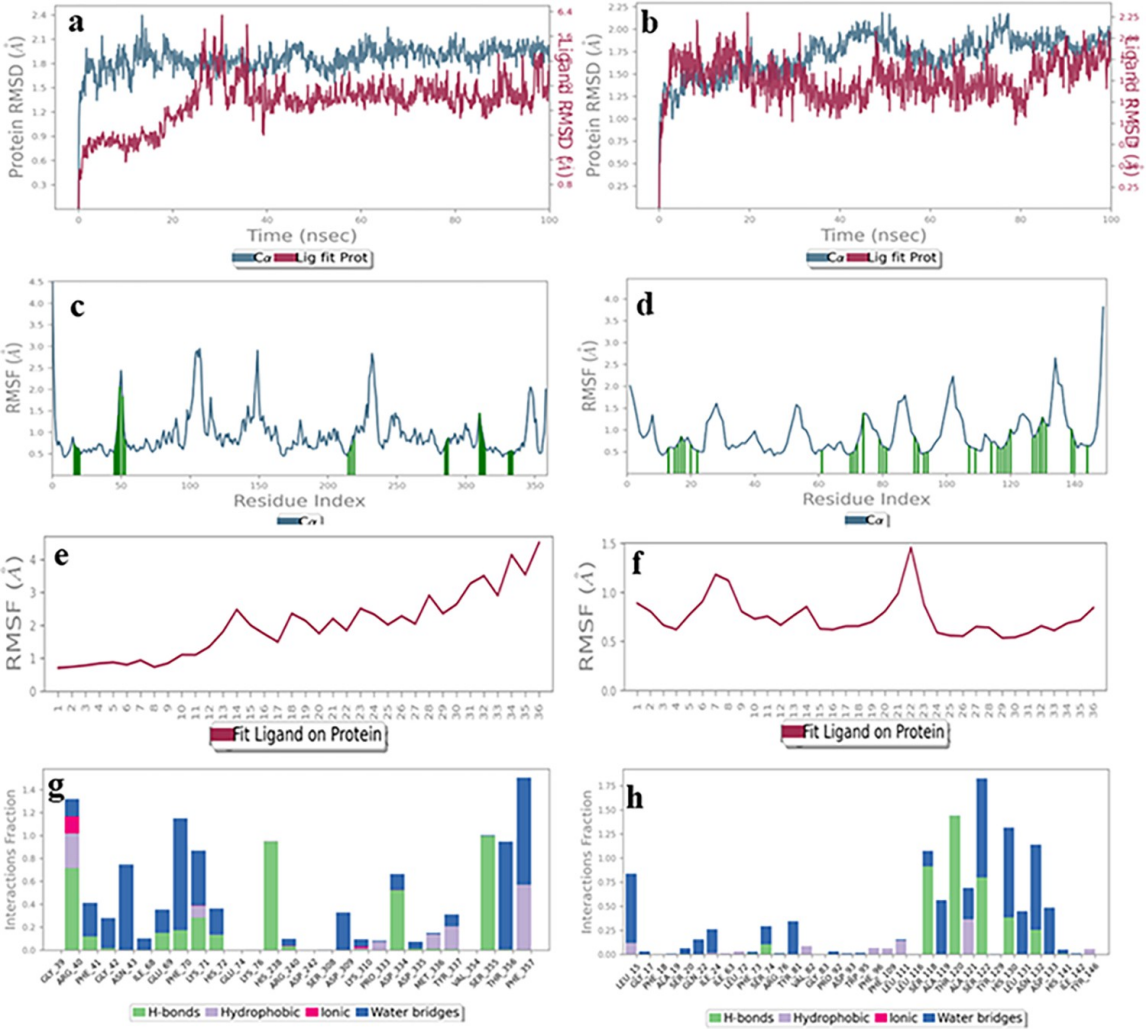
The molecular dynamics trajectories of ceftriaxone sodium with protein acceptor (**a**, **c**, **e**, **g**) and donor binding sites (**b**, **d**, **f**, **h**). **(a, b)** Protein-ligand RMSD **(c, d)** Protein RMSF (**e**, **f**) Ligand RMSF, **(g, h)** Protein-ligand interaction histogram.

The RMSD plot of the ligand-receptor complex for ceftriaxone sodium (**5**) showed less than 3 Å perturbation in protein and ligand structure for both the binding sites (Fig 16A and 16B). Similarly, the RMSF of protein was similar for both binding sites (less than 2.5 Å) (Fig 16C and 16D). However, the ligand RMSF showed a better binding in the acceptor domain (less than 1.5 Å), while it fluctuated relatively more in the donor binding site (more than 3 Å) (Fig 16E and 16F). The interactions of protein with the ligand were significant in both the cases. For instance, in the case of the donor binding domain, Gly39, Asn43, Phe70, Lys71, His238, Asp334, Ser355, Thr356, Phe357 residues interacted *via* hydrogen bonding and water bridge formation, which remained for more than 50% of the simulation time. While, in the acceptor binding domain, Ser118, Al119, Thr120, Ala121, Ser122, His130, Leu131, Asn132, and Asp133 residues were involved in hydrogen bonding and water bridge formation (Fig 16G and 16H). Hence, it can be concluded that ceftriaxone sodium is equally suitable for binding in donor and acceptor binding sites [41].

## 4. Conclusion

Aberrant fucosylation associated with the over-expression of fucosyltransferases (FUTs), is a hallmark of various cancers. Hence, fucosyltransferases (FUTs) have emerged as promising targets for anticancer drug discovery. The current study was an effort to get insights into the structural aspects of fucosyltransferase 2 (FUT2). A drug repositioning strategy was employed, owing to its cost and time effectiveness, in comparison to the conventional drug discovery process. Five drugs [enalaprilat dihydrate, ibuprofen, ascorbic acid, acarbose, and ceftriaxone sodium] showed better docking scores and binding energies against fucosyltransferase 2 (FUT2). Docking and STD-NMR studies were in agreement with each other. The ligand resonances in STD-NMR spectra showed non-covalent interactions as predicted from docking studies. Drugs accommodated better in the donor binding site, and interacted with conserved residues of fucosyltransferase 2 (FUT2), such as Ser355, Phe357, and Thr356. Furthermore, MD simulations for both the donor and acceptor sites of fucosyltransferase 2 (FUT2) also supported the above-mentioned results, as drug- fucosyltransferase 2 (FUT2) complexes were genrelly more stable in the case of the donor binding site, except for ceftriaxone sodium that showed equal probability for both the sites. This study has identified these drugs as initial hits that can be proceeded for lead optimization against fucosyltransferase 2 (FUT2) *via in-vitro*, and pre-clinical studies.

## Supporting information

S1 Table The US FDA approved drugs names, structures, and therapeutic use are given below. (DOCX)

S2 Table Compounds docking scores and binding energy calculation. (DOCX)
