# Supplementary material for: Drug repurposing against fucosyltransferase-2 via docking, STD-NMR, and molecular dynamic simulation studies
Source: PLoS One. 2024 Nov 1;19(11):e0308517. doi: 10.1371/journal.pone.0308517 (PMC11530067; doi:10.1371/journal.pone.0308517)
Supplement: S1 Table — (DOCX) [file pone.0308517.s001.docx]

**Table-S1: The US FDA approved drugs names, structures, and therapeutic uses.**

| **S. No.** | **Drugs Names and Codes** | **Structures** | **Therapeutic Use** |
| --- | --- | --- | --- |
| **1** | **Enalaprilat Dihydrate (1)** |  | Enalapril used in the treatment of hypertension. It is an (Angiotensin Converting Enzyme) ACE inhibitor that prevents (ACE) from transforming angiotensin I into angiotensin II.  <https://pubchem.ncbi.nlm.nih.gov/compound/Enalaprilat-dihydrate>. |
| **2** | **Ibuprofen (2)** |  | Ibuprofen one of a group of painkillers called non-steroidal anti-inflammatory drugs (NSAIDs).  <https://pubchem.ncbi.nlm.nih.gov/compound/Ibuprofen> |
| **3** | **Ceftriaxone Sodium (3)** |  | Ceftriaxone belongs to the class of medicines known as cephalosporin antibiotics. It has a role as an antibacterial drug. It works by killing bacteria or preventing their growth.  <https://doi.org/10.2165/00003495-198427060-00001> |
| **4** | **Ascorbic acid (4)** |  | Ascorbic acid is used to prevent or treat low levels of vitamin C in people who do not get enough of the vitamin from their diets. Low levels of vitamin C can result in a condition called scurvy. It is needed to maintain the health of skin, cartilage, teeth, bone, and blood vessels. It is also used to protect your body's cells from damage. It is known as an antioxidant.  <https://pubchem.ncbi.nlm.nih.gov/compound/Ascorbic-Acid> |
| **5** | **Acarbose (5)** |  | Acarbose is used to treat type 2 diabetes.  <https://doi.org/10.2165/00003495-198835030-00003> |
