# Supplementary material for: Drug repurposing against fucosyltransferase-2 via docking, STD-NMR, and molecular dynamic simulation studies
Source: PLoS One. 2024 Nov 1;19(11):e0308517. doi: 10.1371/journal.pone.0308517 (PMC11530067; doi:10.1371/journal.pone.0308517)
Supplement: S2 Table — (DOCX) [file pone.0308517.s002.docx]

**Table-S2: Compounds docking scores and binding energy calculation.**

| **S. No.** | **Drugs Names and Codes** | **Docking Score** | | **MMGBSA dG Bind (ΔGbind, Kcal/mol)** | |
| --- | --- | --- | --- | --- | --- |
|  |  | **3ZY5** | **1W3F** | **3ZY5** | **1W3F** |
| **1** |   **Acarbose (4)** | -11.013 | -9.362 | -76.06 | -66.95 |
| **2** |   **Enalaprilat Dihydrate (1)** | -9.474 | -5.363 | - 96.46 | - 29.04 |
| **3** |   **Ascorbic acid (3)** | -9.149 | -4.425 | - 60.23 | - 21.12 |
| **4** |   **Ibuprofen (2)** | -7.077 | -2.583 | - 63.02 | -32.09 |
| **5** |   **Ceftriaxone Sodium (5)** | -8.241 | -4.002 | -79.89 | -38.26 |
